# Lack of Induction of RBD-Specific Neutralizing Antibodies despite Repeated Heterologous SARS-CoV-2 Vaccination Leading to Seroconversion and Establishment of T Cell-Specific Memory in a Patient in Remission of Multiple Myeloma

**DOI:** 10.3390/vaccines10030374

**Published:** 2022-02-27

**Authors:** Bernhard Kratzer, Doris Trapin, Pia Gattinger, Teresa Oberhofer, Al Nasar Ahmed Sehgal, Petra Waidhofer-Söllner, Arno Rottal, Ulrike Körmöczi, Katharina Grabmeier-Pfistershammer, Gerhard H. Kopetzky, Franz Tischer, Rudolf Valenta, Winfried F. Pickl

**Affiliations:** 1 Division of Cellular Immunology and Immunohematology, Institute of Immunology, Center for Pathophysiology, Infectiology and Immunology, Medical University of Vienna, 1090 Vienna, Austria; doris.trapin@meduniwien.ac.at (D.T.); teresa.oberhofer@meduniwien.ac.at (T.O.); al.sehgal@meduniwien.ac.at (A.N.A.S.); petra.waidhofer-soellner@meduniwien.ac.at (P.W.-S.); arno.rottal@meduniwien.ac.at (A.R.); ulrike.koermoeczi@meduniwien.ac.at (U.K.); katharina.pfistershammer@meduniwien.ac.at (K.G.-P.); 2 Department of Pathophysiology and Allergy Research, Center for Pathophysiology, Infectiology and Immunology, Medical University of Vienna, 1090Vienna, Austria; pia.gattinger@meduniwien.ac.at (P.G.); rudolf.valenta@meduniwien.ac.at (R.V.); 3 1st Med. Department Hemato-Oncology, Universitätsklinik St. Poelten, 3100 St. Poelten, Austria; gerhard.kopetzky@stpoelten.lknoe.at; 4 Landesklinikum Lilienfeld, 3180 Lilienfeld, Austria; dr.tischer@outlook.com; 5 Laboratory for Immunopathology, Department of Clinical Immunology and Allergology, I. M. Sechenov First Moscow State Medical University (Sechenov University), 119991 Moscow, Russia; 6 NRC Institute of Immunology FMBA of Russia, 115478 Moscow, Russia; 7 Karl Landsteiner University of Health Sciences, 3500 Krems, Austria

**Keywords:** SARS-CoV-2, prophylactic vaccination, multiple myeloma, pomalidomide

## Abstract

Background: Prophylactic vaccination against infectious diseases may induce a state of long-term protection in the otherwise healthy host. However, the situation is less predictable in immunocompromised patients and may require adjustment of vaccination schedules and/or basic therapy. Methods: A patient in full remission of multiple myeloma since the last three years and on long-term maintenance therapy with pomalidomide, a drug inhibiting angiogenesis and myeloma cell growth, was vaccinated twice with Comirnaty followed by two vaccinations with Vaxzevria. Seroconversion and SARS-CoV-2-specific cellular responses were monitored. Results: No signs of seroconversion or T cellular memory were observed after the first “full immunization” with Comirnaty. Consequently, long-term-maintenance therapy with Pomalidomide was stopped and two additional shots of Vaxzevria were administered after which the patient seroconverted with Spike(S)-protein specific antibody levels reaching 49 BAU/mL, mild S-peptide pool-specific T cell proliferation, effector cytokine production (IL-2, IL-13), and T cellular activation with increased numbers of CD3^+^CD4^+^CD25^+^ T cells as compared to vaccinated and non-vaccinated control subjects. However, despite suspension of immunosuppression and administration of in total four consecutive heterologous SARS-CoV-2 vaccine shots, the patient did not develop neutralizing RBD-specific antibodies. Conclusions: Despite immunomonitoring-based adjustment of vaccination and/or therapy schedules vaccination success, with clear correlates of protection, the development of RBD-specific antibodies could not be achieved in the immunocompromised patient with current SARS-CoV-2 vaccines. Thus, our report emphasizes the need for improved active and passive immunization strategies for SARS-CoV-2 infections.

## 1. Introduction

In healthy individuals, SARS-CoV-2 genetic vaccines are generally very successful, inducing high levels of spike protein (S)-specific antibodies in parallel with SARS-CoV-2-specific T-cell memory [1,2,3,4,5]. The induced immunity subsequently protects against severe COVID-19, but booster vaccinations must be given in a timely manner to counteract the consistent decline in antibody levels 4–6 months after completion of the 2-shot immunization cycle and to increase the likelihood of protection against new mutant strains of SARS-CoV-2 [6]. However, the situation is getting more complicated in individuals who have had to undergo B-cell-depleting therapy, e.g., after non-Hodgkin lymphoma, and are thus potentially immunocompromised [7,8,9]. Even patients who have near to normal peripheral blood (PB) counts long after disease remission may still respond poorly or not at all to SARS-CoV-2 genetic vaccines. We describe here the treatment of a patient who developed multiple myeloma and went into full remission 2.5 years before the onset of the actual SARS-CoV-2 pandemic/1.5 years before global vaccination against SARS-CoV-2 was initiated, but whose immune system had significant problems responding to standard SARS-CoV-2 vaccination protocols. In fact, there are reports demonstrating that it is possible to achieve successful seroconversion with two doses of the available SARS-CoV-2 vaccines in multiple myeloma patients in remission [10]. However, little is known about how to manage such patients when they remain non-responsive after a full course of two vaccinations. The patient described in this study is a non-responder to full vaccination with two doses and we report on the outcome of an additional full course of two doses with a heterologous vaccine. Obtained results from this patient highlight the need for improved active or passive immunization strategies for poor responders treated with available COVID-19 vaccines.

## 2. Materials and Methods

### 2.1. Patient Characteristics

**Figure 1:**
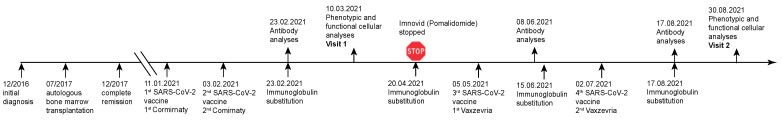
Timeline from diagnosis of multiple myeloma until complete remission of disease and subsequent vaccination course. Relevant timepoints of medical interventions and sampling are indicated.

A 58-year-old patient developed multiple myeloma of the IgG kappa type at the age of 53 (initial diagnosis 12/2016) (Figure 1). The underlying disease was classified as R-ISS II, with 80% infiltration of the bone marrow (BM). The infiltrating cells were CD20^+^ (70%), Cyclin D1^+^ and CD56^+^. Induction was performed with 3 cycles of bortezomib, lenalidomide, and dexamethasone (VRD), but proved to be unsuccessful. Therefore a switch to Carfilzomib-Pomalidomide-Dexamethasone (KPD) paralleled by rituximab treatment was performed, which led to successful stem cell harvest 07/2017 and KPD consolidation post autologous BM transplantation with molecular full remission 12/2017. Long-term consolidation therapy was performed with Imnovid (Pomalidomide, 2 mg/die) from December 2017 until April 2021. Due to therapy-induced hypogammaglobulinemia with IgG levels ranging between 332 mg/dl to 508 mg/dl, the patient received regular immunoglobulin substitution (Privigen, 30 g, i.v.) every other month. Currently, the patient is in sustained complete remission (confirmed by positron emission tomography (PET) in April 2021) with negative minimal residual disease (MRD) (January 2021).

### 2.2. T Cell Proliferation Assays

Peripheral blood mononuclear cells of heparinized venous blood were isolated according to standard protocols [11]. Briefly, fresh heparinized blood was first centrifuged at 500 g for 10 min to obtain plasma and the cellular fraction was resuspended in the double of the original blood volume with IMDM medium (Hyclone, Cytiva, Pasching, Austria) containing 20 U/mL heparin, 10% FCS and antibiotics (15 µg/mL Gentamicin; 0.5 µg/mL Amphotericin). This mixture was overlaid onto Ficoll-Hypaque gradients in 50 mL tubes followed by centrifugation at 500 g for 15 min. The PBMC-rich interphase was collected, washed twice with fresh medium and frozen in IMDM containing 20% FBS and 10% DMSO in liquid nitrogen for subsequent proliferation assays. For T cell proliferation assays, the cells were thawed by gently adding fresh medium dropwise and were subsequentially washed two times. Finally, cells were adjusted to 1 × 10^6^ cells/mL with RPMI 1640 medium (Hyclone, Cytiva, Pasching, Austria) containing 2% human serum (Sigma-Aldrich, St. Louis, MO, USA). The proliferation assays were set up in 96-well round bottom plates (Sarstedt, Nümbrecht, Germany) in triplicates with 1 × 10^5^ cells/well with the different stimuli in a total volume of 200 µL. As stimuli, Tetanus toxoid (12.5 mU/mL, Glaxo Smith Kline), Phytohemagglutinin (PHA, 6.25 µg/mL, Thermo Fisher), Staphylococcal enterotoxin B (SEB, 200 ng/mL, Sigma-Aldrich), pools of S-, S1- and S+- peptide mixes (120 pmol/mL for each peptide, Miltenyi Biotech, Bergisch Gladbach, Germany, consisting of 15-mer sequences with 11 amino acids overlap, covering the sequence of the surface (or spike) glycoprotein (“S”) of SARS-Coronavirus 2 (GenBank MN908947.3, Protein QHD43416.1).), M-peptide-mix (matrix, 120 pmol/mL for each peptide, Miltenyi Biotech, consisting mainly of 15-mer sequences with 11 amino acids overlap, covering the complete sequence of the membrane glycoprotein (“M”) of SARS-Coronavirus 2 (GenBank MN908947.3, Protein QHD43419.1)) or medium alone were used. Cells were incubated for 144 h (6 days) and 100 µL of supernatants were collected from each well and stored frozen at −80 °C for subsequent cytokine determinations (see below). In addition, 100 µL fresh medium was added to obtain a total of 200 µL and cells were pulsed with methyl-[3H]thymidine (1 µCi/well) for 18 h. Finally, T cell proliferation was quantified as a function of the incorporated radioactivity on a Betaplate Counter (Perkin Elmer, Waltham, MA, USA).

### 2.3. Whole Blood Cytokine-Secretion and Cellular Activation Assays

For stimulation of T cells in whole blood (WB), 300 µL of the stimuli Tetanus toxoid (12.5 mU/mL, Glaxo Smith Kline), phytohemagglutinin (PHA, 6.25 µg/mL, Thermo Fisher), Staphylococcal enterotoxin B (SEB, 200 ng/mL, Sigma-Aldrich), and pools of S-, S1- and S+- peptide mixes (120 pmol/mL for each peptide, Miltenyi Biotech, Bergisch Gladbach, Germany), M-peptide-mix (matrix, 120 pmol/mL for each peptide, Miltenyi Biotech) or medium alone were pipetted into sterile 5 mL polystyrene round-bottom tubes (12 mm × 75 mm tubes with caps, BD Biosciences, San Diego, CA, USA). To each 300 µL of stimulus, 300 µL of fresh heparinized WB was added and thoroughly mixed by vortexing three times for one second. Subsequently, cells were incubated with semi-closed lids at 37 °C in a 5% CO_2_ atmosphere at 95% humidity for 44 h. Afterwards, the mixture was again vortexed as described above and tubes were centrifuged at 600 g for 10 min. Next, 300 µL of the cell free supernatant was removed and stored frozen at −80 °C until cytokine analyses. Cells were resuspended by vortexing and 100 µL of the respective cell suspensions from each tube were transferred into fresh tubes for staining and subsequent flow cytometric analyses.

### 2.4. Immunophenotyping of Activated Lymphocytes

Flow cytometric immunophenotyping of leukocytes was performed according to standard quality-controlled (inter-laboratory test validated) procedures [12,13] with the antibodies listed in Table S1. Briefly, 100 µL of serum-free whole blood, washed three times with PBS, was incubated with optimal concentrations of directly conjugated antibodies and incubated at room temperature for 15 min. Subsequentially, 100 µL of Nordic lyse (Nordic MUbio, Susteren, The Netherlands) was added at room temperature for 10 min and afterwards red blood cells were lysed by addition of 4.5 mL of dH_2_O for 5 min. After centrifugation, samples were analyzed on a Navios Ex flow cytometer (Beckman Coulter, Brea, CA, USA) equipped with three laser lines and analyzed with the Kaluza software package (Beckman Coulter, Brea, CA, USA).

For the analysis of T cell activation after incubation of WB with the indicated stimuli, 100 µL aliquots of WB were washed twice (500 g, 5 min) with 4.5 mL of PBS and incubated at room temperature with 1 µL Aqua Zombie viability dye (Biolegend, San Diego, CA, USA) for 10 min. To stop the reaction, cells were washed once with PBS containing 0.5% BSA and 0.05% NaN_3_ and the supernatant was removed. The remaining 100 µL of WB were incubated with the optimal concentrations of antibodies listed in Table S1 at room temperature for 15 min. Finally, cells were fixed and lysed by incubation with 100 µL Nordic lyse (Nordic MUbio, Susteren, The Netherlands) at room temperature for 10 min and incubated with 4.5 mL of dH_2_O for 5 min. The erythrocyte-free samples were acquired on a Navios Ex flow cytometer (Beckman Coulter, Brea, CA) equipped with three laser lines and analyzed with the FlowJo software package (FlowJo LLC, Ashland, OR, USA). Gates were set according to biological controls (stimulation with medium only). Percentages of AIM^+^ T cells were determined for CD4^+^ and CD8^+^ T cells separately and presented as indicated.

### 2.5. Determination of Cytokines in Cell Culture Supernatants

The cytokines released in the supernatants of cultured cells were analyzed by a bead-based multiplex assay (Merck Millipore, Billerica, MA, USA) according to the manufacturer’s recommendations. Briefly, supernatants were thawed at room temperature and 25 µL of the supernatants were incubated with magnetic beads coated with the respective capture antibodies, (anti-human TNF-α, IL-2, IFN-γ, and IL-13), at 4 °C overnight. Subsequently, samples were washed and incubated with biotinylated secondary antibodies at room temperature for 1 h followed by adding streptavidin-PE at room temperature for 30 min. After a final wash, fluorescence intensities of individual bead populations were determined with a Luminex 100/200 apparatus (Luminex corporation, Austin, TX, USA) and were related to standard curves obtained using known cytokine concentrations with the help of which absolute cytokine concentrations were calculated, accordingly.

### 2.6. SARS-CoV-2-Specific Serology

SARS-CoV-2 antibody levels were analyzed as described [14,15] with the following changes. IgG responses to SARS-CoV-2 S protein and the receptor binding domain (RBD) were determined by ELISA. SARS-CoV-2 S protein (Genscript, Piscataway, NJ, USA) and RBD (GenScript) were coated at a concentration of 2 µg/mL in PBS onto NUNC Maxisorb 96 well plates (Thermofisher, Thermo-Fisher Scientific, Waltham, MA, USA) at room temperature overnight. Subsequently, plates were washed three times (PBS, 0.05% Tween 20) and then blocked (PBS, 0.05% Tween 20, 2% BSA) overnight. Serum samples were applied in diluted form (1:50) and incubated at 4 °C for 3 h. For determination of IgG reactivity, plates were washed three times and incubated with HRP-conjugated anti-human IgG (1:1000 diluted, BD, San Jose, CA, USA) for 1 h. After three more washing steps, the bound antibodies were detected with ABTS substrate (Sigma-Aldrich, St. Louis, MO, USA) and the optical density (OD_405_) was determined using an Infinite F50 ELISA reader (Tecan, Männedorf, Switzerland) with 492 nm as the reference wavelength. In addition, SARS-CoV-2 S protein antibody levels were determined three times in an outpatient clinic according to validated methods. On 23 February 2021, an electrochemiluminescence immunoassay (ECLIA) was used, on 8 June 2021 an ELISA benchmarked to the World Health Organization National Institute of Biological Standards (WHO NIBSC) code 20/136 standard with a cut-off value of 15.0 BAU/mL was used, and on 18 August 2021 an electrochemiluminescence immunoassay (ECLIA) from Roche was used with a cut-off value of 0.8 BAU/mL.

### 2.7. Statistics

Statistically significant differences were analyzed with the Kruskal-Wallis test, followed by Dunn’s multiple comparison testing. Ns—not significant, *—*p* < 0.05, **—*p* < 0.01, and ***—*p* < 0.001, respectively.

## 3. Results

### 3.1. Repeated Heterologous Immunization with mRNA- Followed by Vector-Based SARS-CoV-2 Vaccines Leads to Seroconversion, However, without Induction of Neutralizing anti-RBD Antibodies

We here investigated in detail the vaccination response in a patient who suffered from multiple myeloma more than four years ago, achieved full remission more than three years ago, and now underwent full basic immunization (i.e., two vaccinations) with Comirnaty (Tozinameran, BNT162b2), followed by two additional vaccinations with Vaxzevria (ChAdOx1-S). The timeline shows the course of disease, the dates of the vaccinations, Ig-substitutions, venipunctures, and blood cell analyses (Figure 1). The patient received the first immunization with Comirnaty 01/2021, followed by a booster vaccination three weeks later. At that time, the patient was still receiving long-term consolidation therapy with Imnovid (Pomalidomide, 2 mg/day) and regular immunoglobulin infusions in two month intervals (30 g Privigen, Behring, Germany). Five weeks after the second vaccination, peripheral blood mononuclear cells (PBMC) and serum samples were analyzed for cellular and humoral immunity against SARS-CoV-2 (visit 1). The peripheral blood differential showed mild B and T lymphopenia and mild neutropenia (Table 1 and Table S1). Since no humoral immunity was detected at this timepoint, another vaccination course was initiated. Moreover, the patient was advised to stop Imnovid medication in April 2021 because he was in long-term molecular and clinical remission of his primary disease, i.e., multiple myeloma, and the third SARS-CoV-2 vaccination, i.e., the first with Vaxzevria was administered in May 2021. Eight weeks after the first vaccination with Vaxzevria, the fourth SARS-CoV-2 vaccination, i.e., the second dose of Vaxzevria, was administered in July 2021.

**Figure 2:**
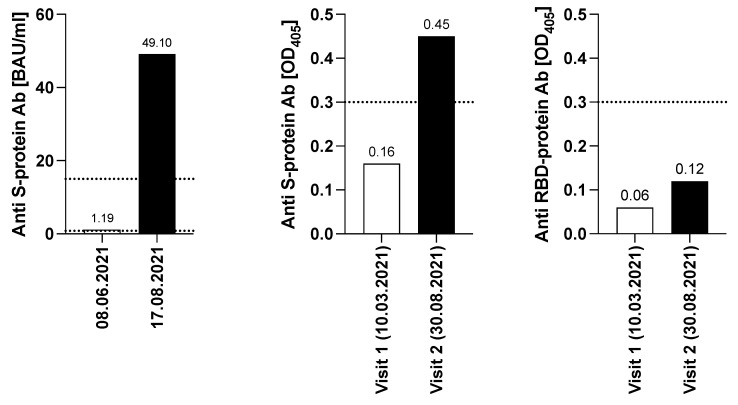
Determination of S- and RBD -specific antibody levels. Y-axes indicate the S- specific BAU/mL levels (left graph, ECLIA, Roche and validated with WHO NIBSC code 20/136) or the OD_405_-values (middle and right graph) obtained with S- or RBD while x-axes show the respective dates of venipuncture. Dotted lines show the cut-off values of the respective tests l. The two dotted lines represent the different cut-off values of the individual test. The cut-off was set at 15.0 BAU/mL for the visit on 8 June 2021, while it was set as 0.8 BAU/mL for the visit on 17 August 2021.

The detailed work-up of the humoral response of the patient after the first two vaccinations with Comirnaty showed neither evidence for the development of a specific humoral immune response against SARS-CoV-2 in an outpatient laboratory test (ECLIA, performed on 23 February 2021), nor in **visit 1 serum** (10 March 2021) [14] detecting S- and RBD-specific serum antibodies, which revealed OD_405_-values of 0.16 and 0.06, respectively, with cut-off OD_405_-values of 0.3. (Figure 2). The patient remained negative after the third vaccination (1.19 BAU/mL; cut-off value 15 BAU/mL on 8 June 2021). The picture slightly changed after completion of the second immunization cycle with the vector vaccine Vaxzevria (ChAdOx1-S). Notably, a value of 49.1 BAU/mL for SARS-CoV-2-specific serum IgG (ECLIA, Roche; 17 August 2021) was detected, which was confirmed by **visit 2 serum** (30 August 2021), (OD_405_ level of 0.45). Despite moderate signs for seroconversion, RBD-specific antibody levels remained negative (OD_405_-value of 0.12). This **visit 2 serum** was also tested for inhibition in a molecular interaction assay (MIA) determining the interaction of SARS-CoV-2 RBD with its cellular receptor ACE2 [14], however, no inhibition of RBD binding to ACE2 was found (not shown). Taken together, the serological results show that repeated vaccinations may lead to a moderate humoral response in the immunocompromised patient, which, in this special case may also have benefitted from stopping the long-term consolidation therapy with Imnovid (Pomalidomide), however, without signs of induction of neutralizing antibodies.

### 3.2. Induction of SARS-CoV-2 Specific T Cell Responses upon Repeated and Cross-Vaccination

**Figure 3:**
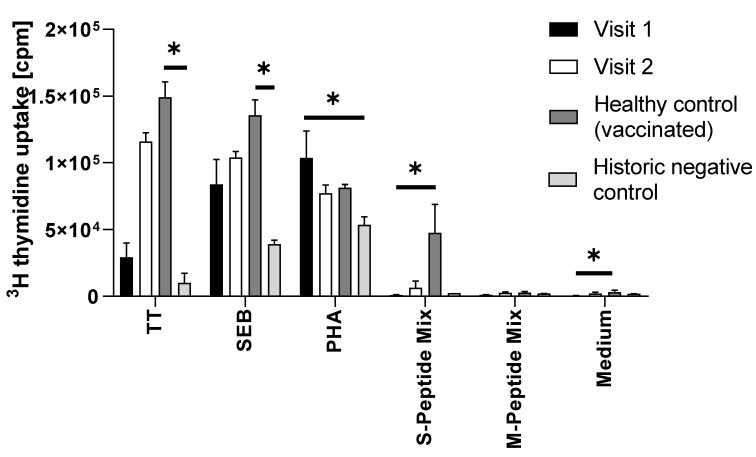
Summary of proliferation results (counts per minute, cpm, *y*-axis) of PBMC that were incubated with the indicated stimuli (*x*-axis). The bars represent the mean and the whiskers the standard deviation of triplicates. Filled bars show the proliferation of PBMC from the immunocompromised patient obtained at visit 1 (after two shots of Comirnaty). Open bars show the proliferative response of PBMC of the patient at visit two (after two additional shots of Vaxzevria), dark grey bars show the proliferation of PBMC of a vaccinated healthy control subject (two shots of Vaxzevria), and light grey bars the response of PBMC of a historic negative control, sampled before the start of the pandemic. TT, tetanus toxoid; SEB, *Staphylococcal* enterotoxin B PHA, phytohemagglutinin; S-peptide mix, SARS-CoV-2 spike protein peptide mix; M-peptide-mix, SARS-CoV-2 matrix protein peptide mix. Only significant differences are shown. *, *p* < 0.05.

The patient’s T cells showed a strong proliferative response upon incubation with tetanus toxoid (Figure 3; stimulation index (SI) 66.5 ± 24.5), demonstrating T cell memory responses to previous vaccine antigens. The polyclonal responses upon incubation with SEB and PHA were also comparable to that of a healthy control subject. Notably, the proliferation upon incubation with SARS-CoV-2 specific antigens modestly increased from visit 1 to visit 2 exclusively in response to the S-specific but not to the M-specific peptide mix (Figure 3). In fact, the SI upon incubation with the S-specific peptide mix changed from 2.1 ± 0.8 to 3.2 ± 2.4 (cut-off for positive proliferation results being a SI of 3.0). In comparison to the response of the PBMC of the vaccinated healthy control individual, (Figure 3), which were obtained 14 weeks after the second vaccination with Vaxzevria, and which revealed an SI of 15.2 ± 6.8 after incubation with S-peptide mix, but not the historic control individual, the patient’s response was weak (SI of only 1.3 ± 0.0). Neither the patient’s PBMC at visit 1 nor at visit 2, nor the PBMC of the healthy control individual nor those of the historic control showed signs of proliferation upon incubation with M-peptide mix (Figure 3).

### 3.3. Analyses of Secreted Cytokines from Proliferation and Whole Blood Assays Point towards the Induction of SARS-CoV-2-Specific Immunity

Next, inflammatory, T helper(h)1 and Th2 cytokines TNF-α, IL-2, IFN-γ, and IL-13 were analyzed in the cell culture supernatants of PBMC incubated with the indicated stimuli (Table S2). Incubation of patient’s PBMC obtained at visit 2 but not at visit 1 with S- but not M-protein mix revealed a 4.1-fold increase in IL-2 and a 4.4-fold increase in IL-13 secretion. Such immune activation was not seen with PBMC of the historic control subject (not shown) (Table S2).

Results obtained with gradient-purified PBMC were corroborated in short-term WB assays. These assays revealed that incubation of WB with SARS-CoV-2 S-protein peptide mix led to a 30.3-fold induction of IL-13, a 3.0-fold induction of IFN-γ and a 6.9-fold induction of TNF-α, while there was no relevant change of IL-2 secretion (Table S3). Compared to the WB responses of the vaccinated control subject (IL-13 39.1-fold, IFN-γ 389.6-fold, TNF-α 40.0-fold, and IL-2 51.5-fold) the patient’s responses were moderate. (Table S3).

### 3.4. Stimulation with S-Protein-Derived Peptide Mix Leads to Specific Activation Induced Marker (AIM) Expression on Patient’s CD4 Helper T Cells

Antigen-specific T cell activation may also cause neo-expression of AIM on the T cell surface (Table S4). Thus, we examined neo-expression of the high-affinity IL-2 receptor CD25 on CD3^+^CD4^+^ Th cells. We found T cell activation in response to the vaccination antigen Tetanus toxoid and the polyclonal stimuli PHA and SEB for both the PBMC of the vaccinated healthy control subject and the patient’s PBMCs. Of note, induction of CD25 expression was also observed upon incubation of the patient’s and vaccinated control subject’s PBMC with S-derived peptide mix, pointing to the establishment of recallable T cellular memory in the patient and the vaccinated healthy control (SI of 5.2 and 23.5). While also visible for other AIM, such as CD69 and CD154 on CD4^+^ T cells, the specific induction was less pronounced (2.1- and 2.8-fold compared to the vaccinated healthy control with 6.4- and 8.0-fold) (Table S4 and Figure S1). In addition, in the healthy control, also a marked induction of CD69 and CD25 on CD8^+^ T cells (SI 99.8 and 98.3) was observable, yielding a positive population. In conclusion, these assays indicated signs of immunity also at the T cellular level, although these were less pronounced compared with the fully immunocompetent vaccinated healthy control individual.

## 4. Discussion

We here describe the attempts to induce SARS-CoV-2-specific humoral, cellular, and cytokine responses by vaccination of a patient, in full remission of multiple myeloma since 4 years due to autologous bone marrow transplantation and consolidation treatment with Pomalidomide until April 2021. It has been reported that a full course of SARS-CoV-2 vaccination consisting of a first and second immunization induced SARS-CoV-2-specific immunity in the majority of vaccinated multiple myeloma patients, albeit to a lower degree than vaccination of healthy subjects [10,16].

However, the case presented by us was different from the previously reported ones. Initial vaccination (2x Comirnaty) of the patient described by us while still receiving Pomalidomide treatment as maintenance therapy after remission of multiple myeloma failed to induce SARS-CoV-2 seroconversion (Figure 1 and Figure 2). This was in contrast to other reports showing that ongoing Pomalidomide treatment did not affect the response [17,18,19]. Common risk factors for vaccination failure such as low B cell counts (<30/µL), current anti-CD38 monoclonal antibody treatment or active disease with more than one treatment line were also absent in our patient [17,20]. Although our patient also underwent rituximab treatment as part of induction therapy for autologous bone marrow transplantation in 2017, he has not received specific B-cell depletion treatment with anti-CD20 therapeutics for the last 3.5 years. Thus, it is more than unlikely that the decreased serum IgG levels still observed as well as the decreased response to SARS-CoV-2 vaccination are related to the previous rituximab treatment, however, we cannot entirely exclude that possibility.

In our patient, two additional shots of Vaxzevria and cessation of the Pomalidomide-based consolidation therapy finally led to a significant increase in peripheral B cell counts, reaching normal values with 300 cells/mm^3^, which was paralleled by the production of SARS-CoV-2 spike protein specific IgG antibodies (49.1 BAU/mL) and a 25% increase in IgG trough levels (from <400 mg/dL to >500 mg/dL), although substitution intervals and doses remained constant. The antibody levels were still in the lower range of those usually detectable upon vaccination of healthy subjects, however, they provided evidence for the expansion of a SARS-CoV-2-specific B cell repertoire through repeated vaccination in combination with temporary stop of anti-neoplastic treatment. The normalization of B cell numbers also suggested that the patient did not suffer from a Rituximab-induced “B cell scar”, but rather of Pomalidomide-induced B cell lymphopenia associated with difficulties in vaccine-induced seroconversion. However, these results also show that “vaccinated” does not necessarily mean “successfully immunized so that sufficient levels of protective antibodies are generated” and underlines the necessity to monitor vaccination success not only in healthy individuals, among which a sizable number of vaccine non-responders would otherwise remain undetected, but especially also in those who have been treated for hematological but also other malignant diseases. In addition, we learn that an almost normal peripheral differential blood count is by no means a guarantee for a good vaccine response. This is demonstrated for the concrete case because seroconversion was not paralleled by significant induction of RBD-specific antibody levels. Thus, it remains questionable if and how well the patient will be protected from (severe) COVID-19 if exposed to SARS-CoV-2. That the patient’s immune system is capable of reacting against microbial cues can be deduced from the fact that strong T cell memory responses upon incubation with tetanus toxoid were apparent, which were paralleled with protective levels of IgG antibodies against this and other vaccine antigens (Table 1). The high levels of antibodies against vaccine antigens tetanus toxoid, hemophilus influenzae B, and pneumococcal polysaccharides at trough serum IgG levels indicates that the patient is producing his own antibodies after receiving basic and booster immunizations in the years 2018–2019.

Another intriguing finding of this study is the fact that the moderate humoral responses were strictly accompanied by T cellular memory responses, which is in accordance with previous findings, that in the majority of vaccine non-responders also T cell responses are absent [21]. While it could have been assumed that T cell responses could have established themselves even in the absence of functional B cells, this was not what we found here. Indeed, signs of T cell recall responses coincided with the time point that seroconversion could also be detected, which might speak for an important role of B cells for the induction of recallable T cell memory [22,23]. Furthermore, our results show that it is important not to solely rely on the determination of the obvious effector cytokine, which would be IFN-γ in the case of classic vaccine responses, but also take other effectors such as IL-2 and IL-13 into consideration, which showed moderate but significant S-peptide specific induction in the healthy control subject but also in the patient discussed herein. Building-up of T cellular memory was also observed by determining AIM expression such as CD25 and to a lesser extent CD69 and CD154 on CD3^+^CD4^+^ T cells of this patient and to an even stronger extent on CD3^+^CD4^+^ T cells of the healthy control subject. In contrast, while CD3^+^CD8^+^ T cells of the control subject strongly neo-expressed CD25 and CD69 upon incubation with S-peptide mix, this was not evident with CD3^+^CD8^+^ T cells of the patient, indicating a potential scar in the respective repertoire. Recent reports confirmed these findings, showing that more patients and healthy controls mounted an efficient CD4+ T cell as compared to a CD8+ T cell response [21].

We cannot entirely exclude that the observed moderate S-specific IgG levels could have been passively transferred by the regular immunoglobulin substitution of the patient. However, several points argue against it: (i) patient serum was collected at the trough levels of substituted immunoglobulins, making it unlikely that transfused S-specific IgG have been maintained at such high levels; (ii) no RBD-specific antibody levels whatsoever were detectable in the patient’s serum, which would have to be expected when hyperimmunoglobulin of healthy vaccinees was transfused [24].; (iii) signs for the induction S-specific T cell immune responses became detectable in the patient after the fourth vaccination and coincided with the appearance of specific IgG antibodies, suggesting at least a partial vaccination success.

The encouraging part of this study was that, in principle, it is possible to induce at least the first signs of humoral and cellular immunity even in patients with a compromised immune system by (i) repeated antigen delivery; (ii) changing the formulation of the antigen (mRNA versus vector-based SARS-CoV-2 vaccine), (iii) tapering potentially immunosuppressive therapies if deemed indicated, and (iv) strict immuno-monitoring of the vaccination success, which must guide the three previous parameters on the humoral and cellular level, in order to be able to appreciate the depth of future protection. The discouraging part of the study is certainly the finding that despite four shots with currently available SARS-CoV-2 vaccines, cross-vaccination and shortened vaccination intervals, only very low levels of S-specific antibodies could be induced, while RBD-specific neutralizing antibodies remained completely absent, seriously questioning the further management of such patients with available vaccines. It is well known that neutralizing antibodies, preventing SARS-CoV-2 from docking to its cellular receptor ACE2, are instrumental in protecting from infection and severe disease course. The latter has been convincingly demonstrated by the timely infusion of monoclonal anti-S-protein-specific antibodies into patients, which prevented them from entering into a severe disease course [25,26]. A similar form of passive immunization may be advisable for the patient in question when infected. Alternatively, transfusion of hyperimmunoglobulin of selected plasma donors containing high levels of virus-neutralizing anti-RBD antibodies may be practicable, since the patient was, at least until recently, dependent on general immunoglobulin infusion [27] due to treatment-induced hypogammaglobulinemia.

While in the given patient signs for vaccination-induced immune responses have been achieved by applying four vaccine shots within a relatively short time window of six months, the final aim of inducing high-titer RBD-specific antibody levels able to neutralize spike protein interaction with its cellular receptor ACE2 has been clearly missed despite all efforts. Further escalation of the current vaccination scheme with the currently available vaccines may be futile, thus the development and application of better-targeted and adjuvanted vaccines may be warranted, which also for this patient group may pave the way for a safer life. There are currently more than 300 SARS-CoV-2-specific vaccines in preclinical or clinical development [28] but our case demonstrates that also other strategies such as passive immunization must be considered for treatment of non- or poor responders to active immunization.

## 5. Conclusions

Our report shows that immunomonitoring-based adjustment of vaccination and/or therapy schedules can lead to seroconversion even in the immunocompromised patient, but may still fail to induce protective neutralizing anti-RBD-based immunity.

## Figures and Tables

**Table 1:** Laboratory findings at the two different timepoints of venipuncture of the patient.

	Visit 1 w/Pomalidomide	Visit 2 w/o Pomalidomide	Reference Values
Leukocytes, cells/µL	3300 *	3400	3880–10,640
Granulocytes, cells/µL	1650	1730	2020–8220
Monocytes, cells/µL	660	370	220–990
Lymphocytes, cells/µL	990	1290	1000–2800
CD3^+^ T cells, cells/µL	630	650	700–2100
CD3^+^CD4^+^ T cells, cells/µL	300	260	300–1400
CD3^+^CD8^+^ T cells, cells/µL	270	320	200–900
CD4/CD8 Ratio	1.11	0.80	1.00–3.60
CD19^+^ B cells, cells/µL	70	300	100–500
CD56^+^ CD16^+^ NK cells, cells/µL	240	320	90–600
Anti-S Protein levels (OD_405_)	0.16	0.45	≥0.3
Anti-S Protein levels (BAU/mL)	1.19 **	49.10 ***	≥15 **; ≥0.8 ***
Tetanus toxoid antibodies (IU/mL)	1.08	0.94	0.05–39.62
Diphtheria toxoid antibodies (IU/mL)	0.15	0.08	>0.01
Hemophilus Influenzae B (mg/l)	1.43	1.94	0.09–19.5
Pneumococcal polysaccharide (mg/mL)	33.5	42.4	10–191.2

* Pathological/negative reference values are shown in red font. ** Analyzed on 8 June 2021. *** Analyzed on 17 August 2021.

## Data Availability

Data are obtainable from the authors upon request.

## Supplementary Materials

The following supporting information can be downloaded at: https://www.mdpi.com/article/10.3390/vaccines10030374/s1, Figure S1: Activation-induced marker (AIM) expression on CD4^+^ and CD8^+^ T lymphocytes, Table S1: List of antibodies used in the study, Table S2: Absolute cytokine levels in the supernatants of gradient-isolated PBMC incubated with the indicated antigen-specific and polyclonal stimuli; Table S3. Absolute cytokine levels in the supernatants of whole blood incubated with the indicated anti-gen-specific and polyclonal stimuli; Table S4. Percent activated T cell subsets as determined after incubation with the indicated stimuli.
