# Supplementary material for: Lack of Induction of RBD-Specific Neutralizing Antibodies despite Repeated Heterologous SARS-CoV-2 Vaccination Leading to Seroconversion and Establishment of T Cell-Specific Memory in a Patient in Remission of Multiple Myeloma
Source: Vaccines (Basel). 2022 Feb 27;10(3):374. doi: 10.3390/vaccines10030374 (PMC8949333; doi:10.3390/vaccines10030374)
Supplement: Supplementary file 1 [file vaccines-10-00374-s001.zip › vaccines-1579816-supplementary.pdf]

Supplementary Materials:

List of Supplementary Figures and Tables:

Figure S1: Activation-induced marker (AIM) expression on CD4+ and CD8+ T lymphocytes,

Table S1: List of antibodies used in the study;

Table S2: Absolute cytokine levels in the supernatants of gradient-isolated PBMC incubated with the indicated antigen-specific and polyclonal stimuli;

Table S3. Absolute cytokine levels in the supernatants of whole blood incubated with the indicated antigen-specific and polyclonal stimuli;

Table S4. Percent activated T cell subsets as determined after incubation with the indicated stimuli;

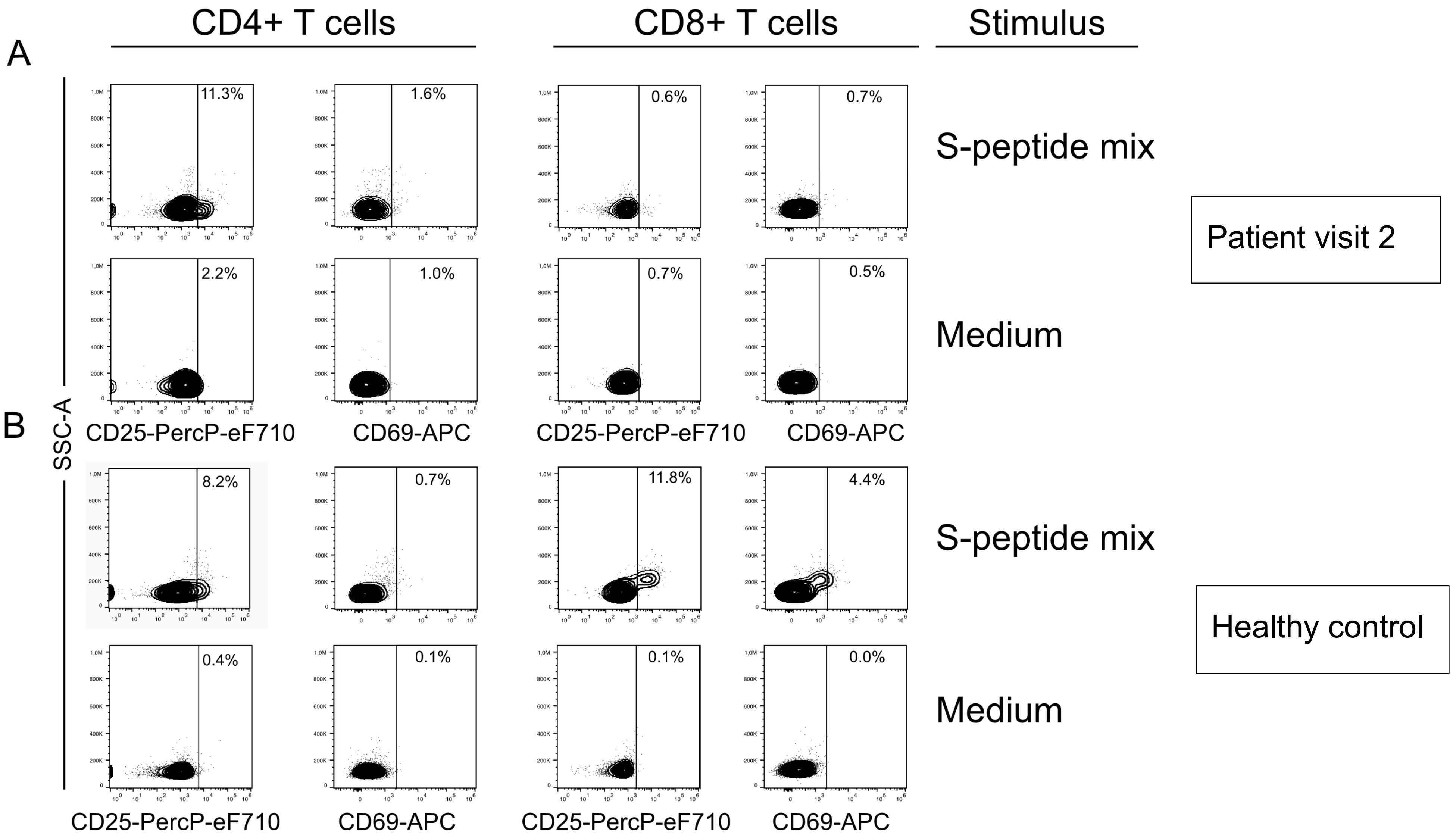

Figure S1. Activation-induced marker (AIM) expression on CD4⁺ and CD8⁺ T lymphocytes. Shown are representative dot plots depicting side scatter (Y-axes) and CD25 or CD69 expression as indicated (X-axes) after incubation with the indicated stimuli. (**A**) Shown are the flow cytometric analyses of the immunocompromised patient at visit 2, (**B**) shown are the flow cytometric analyses of the vaccinated healthy control subject after 44 hours of stimulation. Vertical gates were set according to the staining of cells incubated in medium alone. Numbers indicate percent positive cells within gates.

Table S1. List of antibodies used in the study.

Specificity of	Clone Name	Species	Fluorophore	Source
CD3	UCHT1	Mouse IgG1	PE-Cy7	Thermo Fisher Scientific, Waltham, MA, USA
CD4	RPA-T4	Mouse IgG1	PE-eFluor610	Thermo Fisher Scientific
CD8	RPA-T8	Mouse IgG1	AF700	Thermo Fisher Scientific
CD19	HIB19	Mouse IgG1	eFluor506	Thermo Fisher Scientific
CD25	CD25-4E3	Mouse IgG2b	PerCP-eFluor710	Thermo Fisher Scientific
CD69	FM50	Mouse IgG1	APC	Thermo Fisher Scientific
CD154	24-31	Mouse IgG1	FITC	Thermo Fisher Scientific
HLA-DR	LN3	Mouse IgG2b	eFluor450	Thermo Fisher Scientific
CD8	RPA-T8	Mouse IgG1	FITC	Thermo Fisher Scientific
sIgK	TB28-2	Mouse IgG1	FITC	Thermo Fisher Scientific
TCR a/b	WT31	Mouse IgG1	PE	Thermo Fisher Scientific
CD19	HIB19	Mouse IgG1	PE-eFluor610	Thermo Fisher Scientific
CD33	WM-53	Mouse IgG1	PerCP-Cy5.5	Thermo Fisher Scientific
CD5	L17F12	Mouse IgG2a	APC	Thermo Fisher Scientific
CD20	2H7	Mouse IgG2b	AF700	Thermo Fisher Scientific
CD38	HIT2	Mouse IgG1	APC-eFluor780	Thermo Fisher Scientific
CD45	HI30	Mouse IgG1	eFluor506	Thermo Fisher Scientific

Table S2. Absolute cytokine levels in the supernatants of gradient-isolated PBMC incubated with the indicated antigen-specific and polyclonal stimuli.

Stimulus	Individual	TNF-α [pg/ml]	TNF-α [SI]	IL-2 [pg/ml]	IL-2 [SI]	IFN-γ [pg/ml]	IFN-γ [SI]	IL-13 [pg/ml]	IL-13 [SI]
Tetanus toxoid	Patient visit 1	901.6	0.8	1.0	1.0	153.3	8.7	727.6	485.1
	Patient visit 2	1853.5	2.4	5.1	1.3	2035.1	223.6	2521.6	382.1
	Healthy vaccinated control	2705.6	8.9	8.4	0.6	7017.4	649.8	5488.0	1444.2
Staphylococcal enterotoxin B	Patient visit 1	3435.5	2.9	1.0	1.0	23479.1	1326.5	8252.8	5501.9
	Patient visit 2	4637.5	5.9	0	0.0	>32500.0	>32500.0	7987.2	1210.2
	Healthy vaccinated control	4724.2	15.6	6.2	0.4	148503.8	13750.4	2097.4	551.9
Phytohemagglutinin	Patient visit 1	5221.0	4.4	49.9	49.9	22732.1	1284.3	14340.0	9560.0
	Patient visit 2	6740.6	8.6	9538.3	2384.6	17745.4	<A1950.0	16988.6	2574.0
	Healthy vaccinated control	6368.6	21.0	18293.7	1261.6	126070.0	11673.1	14806.9	3896.6
SARS-CoV-2 spike protein peptide mix	Patient visit 1	1072.8	0.9	1.0	1.0	4.5	0.3	3.0	2.0
	Patient visit 2	774.9	1.0	16.5	4.1	7.4	0.8	29.0	4.4
	Healthy vaccinated control	397.4	1.3	44.4	3.1	181.5	16.8	259.9	68.4
SARS-CoV-2 matrix protein peptide mix	Patient visit 1	999.1	0.8	1.0	1.0	4.5	0.3	1.0	0.7
	Patient visit 2	1374.8	1.8	18.5	4.6	< 0	<0	9.1	1.4
	Healthy vaccinated control	284.8	0.9	9.4	0.6	9.1	0.8	6.6	1.7
Medium	Patient visit 1	1177.4	1.0	1.0	1.0	17.7	1.0	1.5	1.0
	Patient visit 2	783.5	1.0	4.0	1.0	9.1	1.0	6.6	1.0
	Healthy vaccinated control	303.6	1.0	14,5	1,0	10.8	1.0	3.8	1.0

Table S3. Absolute cytokine levels in the supernatants of whole blood incubated with the indicated antigen-specific and polyclonal stimuli

Stimulus	Individual	TNF-α [pg/ml]	TNF-α [SI]	IL-2 [pg/ml]	IL-2 [SI]	IFN-γ [pg/ml]	IFN-γ [SI]	IL-13 [pg/ml]	IL-13 [SI]
Tetanus toxoid	Patient visit 2	91.8	5.1	329.8	329.8	1195.5	209.7	351.7	1172.3
	Healthy vaccinated control	583.3	15.2	2981.5	2981.5	7971.2	514.3	705.6	106.9
Staphylococcal enterotoxin B	Patient visit 2	2082.5	115.1	7068.2	7068.2	14295.5	2508.0	2372.2	7907.3
	Healthy vaccinated control	1917.4	49.8	6221.1	6221.1	15122.8	975.7	844.9	128.0
Phytohemagglu tinin	Patient visit 2	2546.3	140.7	4882.3	4882.3	8827.2	1548.6	3692.5	12308.3
	Healthy vaccinated control	2733.4	71.0	6728.2	6728.2	14251.8	919.5	1498.4	227.0
SARS-CoV-2 spike protein peptide mix	Patient visit 2	124.0	6.9	1.0	1.0	17.1	3.0	9.1	30.3
	Healthy vaccinated control	1540.8	40.0	51.5	51.5	6039.2	389.6	258.3	39.1
SARS-CoV-2 matrix protein peptide mix	Patient visit 2	25.8	1.4	1.0	1.0	4.0	0.7	0.3	1.0
	Healthy vaccinated control	57.1	1.5	0.0	0.0	17.8	1.1	11.4	1.7
Medium	Patient visit 2	18.1	1.0	1.0	1.0	5.7	1.0	0.3	1.0
	Healthy vaccinated control	38.5	1.0	1.0	1.0	15.5	1.0	6.6	1.0

Table S4. Percent activated T cell subsets as determined after incubation with the indicated stimuli.

Stimulus	Individual	CD25$^+$ CD4$^+$ T cells [%] (SI)		CD69$^+$ CD4$^+$ T cells [%] (SI)		CD154$^+$ CD4$^+$ T cells [%] (SI)		HLA-DR$^+$ CD4$^+$ T cells [%] (SI)		CD25$^+$ CD8$^+$ T cells [%] (SI)		CD69$^+$ CD8$^+$ T cells [%] (SI)		CD154$^+$ CD8$^+$ T cells [%] (SI)		HLA-DR$^+$ CD8$^+$ T cells [%] (SI)	
Tetanus toxoid	Visit 2	22.3	(10.2)	6.4	(6.7)	16.9	(15.4)	24.8	(3.7)	7.2	(9.9)	2.7	(5.5)	4.0	(16.7)	27.8	(2.1)
	Healthy control	8.1	(23.2)	1.9	(17.5)	2.1	(23.3)	11.2	(3.0)	3.0	(25.3)	8.6	(194.5)	3.2	(81.8)	19.6	(6.3)
Staphylococcal enterotoxin B	Visit 2	36.7	(16.8)	23.9	(24.9)	44.3	(40.3)	33.2	(4.9)	18.2	(25.3)	29.4	(61.3)	28.2	(117.5)	64.7	(4.8)
	Healthy control	16.6	(47.4)	8.9	(80.9)	7.4	(83.8)	17.5	(4.7)	13.2	(110. 0)	12.5	(284.1)	1.7	(43.8)	24.3	(7.9)
Phytohemagglutinin	Visit 2	66.2	(30.2)	66.7	(69.5)	61.8	(56.2)	56.6	(8.4)	58.4	(81.1)	50.3	(104.8)	28.0	(116.7)	76.0	(5.7)
	Healthy control	72.2	(206.3)	46.5	(422.7)	52.5	(596.6)	34.1	(9.2)	86.9	(724.2)	40.1	(911.4)	10.8	(276.9)	35.8	(11.6)
SARS-CoV-2 spike protein peptide mix	Visit 2	11.3	(5.2)	2.0	(2.1)	3.1	(2.8)	11.4	(1.7)	0.6	(0.8)	0.7	(1.5)	0.4	(1.7)	11.4	(0.9)
	Healthy control	8.2	(23.5)	0.7	(6.4)	0.7	(8.0)	10.6	(2.9)	11.8	(98.3)	4.4	(99.8)	0.4	(10.5)	12.6	(4.1)
SARS-CoV-2 matrix protein peptide mix	Visit 2	3.5	(1.6)	0.7	(0.7)	0.5	(0.5)	8.3	(1.2)	0.2	(0.2)	0.2	(0.3)	0.0	(0.0)	9.8	(0.7)
	Healthy control	2.4	(6.9)	0.1	(0.9)	0.1	(1.1)	5.1	(1.4)	0.3	(2.4)	0.3	(6.6)	0.0	(1.1	4.6	(1.5)
Medium	Visit 2	2.2	(1.0)	1.0	(1.0)	1.1	(1.0)	6.7	(1.0)	0.7	(1.0)	0.5	(1.0)	0.2	(1.0)	13.4	(1.0)
	Healthy control	0.4	(1.0)	0.1	(1.0)	0.1	(1.0)	3.7	(1.0)	0.1	(1.0)	0.0	(1.0)	0.0	(1.0)	3.1	(1.0)
